# Novel Matrix Metalloproteinase-9 (MMP-9) Inhibitors in Cancer Treatment

**DOI:** 10.3390/ijms241512133

**Published:** 2023-07-28

**Authors:** Zainab Ahmed Rashid, Sanaa K. Bardaweel

**Affiliations:** Department of Pharmaceutical Sciences, School of Pharmacy, University of Jordan, Amman 11942, Jordan

**Keywords:** MMP-9, matrix metalloproteinase, inhibitors, molecular docking, anticancer

## Abstract

Matrix metalloproteinases (MMPs) belong to a family of zinc-dependent proteolytic metalloenzymes. MMP-9, a member of the gelatinase B family, is characterized as one of the most intricate MMPs. The crucial involvement of MMP-9 in extracellular matrix (ECM) remodeling underscores its significant correlation with each stage of cancer pathogenesis and progression. The design and synthesis of MMP-9 inhibitors is a potentially attractive research area. Unfortunately, to date, there is no effective MMP-9 inhibitor that passes the clinical trials and is approved by the FDA. This review primarily focuses on exploring the diverse strategies employed in the design and advancement of MMP-9 inhibitors, along with their anticancer effects and selectivity. To illuminate the essential structural characteristics necessary for the future design of novel MMP-9 inhibitors, the current narrative review highlights several recently discovered MMP-9 inhibitors exhibiting notable selectivity and potency.

## 1. Introduction

Cancer is an abnormal cell growth that accounts for a significant portion of global deaths. It is considered the second major cause of death after heart disease [1]. Interestingly, anticancer drug development research focuses on over-expressed molecules that have critical roles in cell survival and proliferation. For instance, the extracellular matrix (ECM) has a major role in cancer-related functions, such as cell cycle regulation, survival, and apoptosis [2]. ECM consists of several molecules, such as proteoglycans, glycosaminoglycans, structural proteins (collagen and elastin), adhesion proteins (fibronectin and laminin), and proteases called matrix metalloproteases (MMPs) [3]. Supportive evidence is available on the key role of the extracellular proteinases (MMPs) as potential modulators of cell–cell and cell–ECM communication, which controls essential tissue homeostasis [4].

MMPs belong to a family of zinc-dependent endopeptidases that consists of 23 members. They participate in several biological and physiological processes and are highly regulated by cytokines, hormones, and growth factors [5]. MMPs have been classified according to their sub-cellular distribution and selectivity for ECM components. The first group includes collagenases (MMP-1, MMP-8, MMP-13, and MMP-18) that are responsible for the degradation of essential components in the bone called helical fibrillar collagen. The second group includes gelatinases (MMP-2 and MMP-9), which are essential in angiogenesis and neurogenesis. Additionally, stromelysins (MMP-3, MMP-10, and MMP-11) and matrilysins (MMP-7 and MMP-26) digest segments of the ECM [3,6]. MMPs are classified by domain structure into eight groups, five of which are secreted and three of which are membrane-associated [3].

Matrix metalloproteinase-9 (MMP-9) is one of the most complex MMPs that belongs to the gelatinase B family [7]. It is mainly located in the hippocampus, cerebellum, and cerebral cortex [8]. It is secreted in two forms, either as zymogens or as an inactive enzyme, from endothelial cells, leukocytes fibroblasts, neutrophils, and macrophages. During granulocyte differentiation, bone marrow is the main site of MMP-9 synthesis [9]. MMP-9 is engaged in several pathophysiological processes, such as extracellular matrix (ECM) degradation, tissue remodeling, and normal tissue turnover. The contribution of MMP-9 in the progression of several diseases was reported in extracranial arteriovenous malformations (AVMs) [10], rheumatoid arthritis [11,12], several neurological diseases and inflammatory processes [13,14], cancer [15,16,17], and ischemic stroke [18,19]. The suppression of MMP-9 activity is achieved by the binding of matrix metalloproteinase inhibitors (MMPIs) to the zinc (Zn^2+^) ion at the catalytic site [20]. It is still not clear how MMPIs suppress cancer growth. Several reports suggest that MMPIs may inhibit cell proliferation by inducing apoptosis through the release of ligands, such as TNFa and TRAIL (Tumor necrosis factor-related apoptosis-inducing ligand), from their membrane-bound inactive form [21].

In recent decades, numerous studies revealed the critical role of MMP-9 in cancer development and progression [18,22,23]. This gelatinase revealed a key role in tumorigenesis through the regulation of several processes, such as the survival of cancer cells, migration, stimulation of immune response, and generation of cancer microenvironment. Consequently, it has become a potentially attractive target for antitumor therapy. Several MMP-9 inhibitors have been synthesized and evaluated for biological activities [24,25,26]. However, the high homology of MMP-9 with other members of MMPs makes the development of effective, selective, and safe MMP-9 inhibitors extremely challenging.

Interestingly, MMPs have been applied in different disciplines of expertise, including biochemistry, cell biology, pathology, immunology, and computational biology. From this perspective, we review the published research regarding the potential role of MMP-9 in cancer development, paying special attention to the computational and synthetic approaches utilized for the design and development of MMP-9 inhibitors.

## 2. Structure and Function

In humans, MMP-9 can be synthesized by and released from neutrophils, macrophages, fibroblasts, and endothelial cells [27]. It is synthesized as a pre-proenzyme and then transferred into the extracellular environment in the form of a pro-MMP-9 enzyme. The activated MMP-9 is produced by the protease-mediated cleavage of the pro-MMP-9 enzyme [27,28]. MMP-3 is an example of those proteases that activate MMP-9 through the removal of the N-terminal pro-peptide region [29]. The removal of the N-terminal pro-peptide significantly disrupts MMP latency.

**Figure 1:**
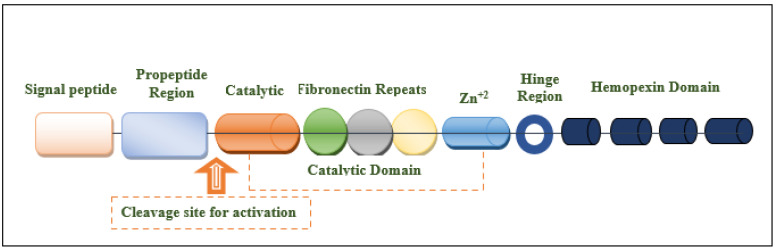
Structural illustration of the domain structures and motifs of MMP-9.

The MMP-9 gene is located on chromosome 20q13.12, which contains 13 exons and 12 introns. This protein is composed of the following domains: signal peptide domain, pro-peptide region, catalytic domain, hemopexin-like domain, and hinge region [27,30,31] (Figure 1). The signal peptide domain is composed of 17–29 amino acids and is responsible for the secretion of MMP-9 [32]. The pro-peptide domain contains 77–87 amino acids. The amino acid sequence of the pro-domain is PRCGXPD. A cystein switch is vital to MMP-9 activation [33,34]. One cysteine residue (cys99) of pro-MMP-9 interacts with the catalytic zinc ion of this protein. This binding is essential for MMP latency [15]. Consequently, MMP-9 activity is suppressed by preventing the binding of water molecules to the zinc ion in the catalytic domain [35]. Another important domain that is responsible for the proteolytic activity of MMP-9 is the catalytic domain. It is structurally spherical and composed of 170 amino acids [36,37]. It contains an essential consensus zinc-binding sequence (HEXXHXXGXXH) for it is potential activity [38]. For structural integrity and specific activity, two zinc ions are available in the catalytic domain maintaining catalytic and structural roles [35]. Additionally, for enzyme stability, five calcium ions are placed in the catalytic domain [32,39]. Sub-domains are found in the catalytic domain, including the N-terminal domain and the C-terminal domain, which are separated by a shallow catalytic cleft and linked with a U loop [39]. The MMP-9 activation process through the coordination with the catalytic zinc ion requires water molecules in addition to three histidine amino acid residues (His218, His222, and His228) [39]. Six binding pockets are present in the catalytic cleft. Three of them are located on the left side (S1, S2, and S3 pockets). On the other hand, S1′, S2′, and S3′ pockets are located on the right side of the catalytic zinc ion [40]. Substrate selectivity is highly dependent on the depth, length, and amino acid sequence of the S1 pocket that varies among different MMPs. MMP-9 is characterized by an intermediate S1 pocket [41]. Comparable size, position in the catalytic domain, and exposure to the solvent of the S1 pocket were shown in both MMP-2 and MMP-9. The only difference was shown in the residues 425–431 that form a loop in MMP-9 but is absent in MMP-2 [19,42,43]. The fibronectin domain is unique to MMP-2 and MMP-9. This domain is composed of three fibronectin type II motifs, inserted into the metalloproteinase domain [35,44,45]. It is considered an essential modulator of gelatin, laminin, and collagens type I and IV recognition, binding, and degradation [40,46,47]. The hemopexin domain is an ellipsoidal-shaped domain, composed of 210 amino acids [30]. The wild-type enzyme contains four blades connected through a disulfide bond between the first and fourth blades [35]. It is an important domain to bind to tissue metalloproteinase inhibitors [15]. The Hinge region is characterized by its flexibility, which confers the mobility between the hemopexin-like domain and the catalytic domain that is essential for enzyme activity [27,48].

Extracellular matrix (ECM) remolding is one of the main functions of MMP-9. It involves the proteolytic cleavage of the most important MMP-9 substrates, including gelatin, elastin, and collagen [49]. In addition, other substrates are specifically cleaved by MMP-9, such as plasma membrane proteins, extracellular proteins, and intracellular proteins [50,51].

Due to this proteolytic cleavage ability of the MMP-9, it is engaged in various biological processes, such as the alteration of cell–cell and cell-ECM interactions [52,53]. Additionally, as collagen type IV is the main constituent in the basement membrane, MMP-9 plays an essential role in its degradation [54,55]. Consequently, tumor cell invasion and metastases are generally enhanced because of basement membrane destruction.

Angiogenesis is essential for tumor cell growth and development. MMP-9 endorses angiogenesis by degrading the basement membrane and ECM component. Consequently, endothelial cell migrates to produce new blood vessels. On the other hand, MMPs may prevent the mechanism of angiogenesis. It causes the release of angiostatin as a result of the degradation of plasminogen and cleaves the collagen XVIII to produce endostatin. MMP-9 helps in the degradation of plasminogen to produce angiostatin, which increases apoptosis in tumor cells [56].

MMP-9 has both pro- and anti-apoptotic activity. The pro-apoptotic activity is due to the alteration of ECM composition. Conversely, the anti-apoptotic activities are due to the cleavage of Fas ligand, activation of protein kinase B, or threonine kinase AKT [56,57].

MMP-9 has a very important role in various neurological and neurodegenerative diseases through ECM degradation, disruption of blood–brain barrier (BBB), and inflammation [58]. In addition, it is also associated with the pathogenesis of epilepsy by reducing synaptic plasticity and the formation of epileptic foci [7].

Moreover, MMP-9 has an important role in acute and chronic inflammatory diseases because it has both pro- and anti-inflammatory effects. It enhances leukocyte influx and BBB permeability, which promotes inflammation. Interestingly, MMP-9 inhibitors may be helpful in some autoimmune diseases, such as rheumatoid arthritis [59].

## 3. Role in Cancers

The extracellular environment is highly involved in several carcinogenic events, including angiogenesis, invasion, and metastasis [60]. The vital involvement of MMP-9 in extracellular matrix (ECM) remodeling unveils a substantial correlation between MMP-9 and each stage of cancer pathogenesis and progression [61,62,63]. MMP-9 has become a highly valuable target that is involved in cancer and many other diseases, such as autoimmune and cardiovascular diseases [64,65,66].

Tumor cells stimulate the surrounding cells to increase the production of MMPs by enhancing the secretion of interferon, interleukins, growth factors, and especially the extracellular MMPs [6]. The overexpression of MMPs is associated with ECM remodeling and the subsequent release of growth factors, which serve as the ideal microenvironment for tumor survival and spreading [44].

The activation of the cell signaling pathway was found to be directly associated with the binding of MMP-9 with surface receptors. Subsequently, major biological events are affected, including cell growth, migration, and survival [67]. Notably, the binding of proMMP-9′s PEX domain to its receptors, including α4, β1 integrin, and CD44, induces an intracellular signaling pathway that enhances the survival of CLL cells. Accordingly, a high level of proMMP-9 is found in CLL cells from blood and lymphoid organs [68]. Additionally, it was found that MMP-9-dependent migration involves the heterodimerization of the PEX domain of proMMP-9 with CD44. This binding leads to the subsequent activation of the tyrosine kinase epidermal growth factor receptor (EGFR) and the further phosphorylation of its downstream kinase effectors ERK, AKT, and FAK (focal adhesion kinase) [69].

Interestingly, the SDS-PAGE zymography assay revealed that gelatinase B plays an essential role in the progression of gastric cancer [70]. Another study revealed that MMP-9 polymorphism has an important role in breast cancer and may help to identify individuals with high risk [71]. Furthermore, it is found that poor prognosis in breast cancer is highly correlated to MMP-9 expression level [72]. The overexpression of avb6 integrin was found in colon cancer, which consequently enhances MMP-9 secretion, followed by protein–kinase-c pathway activation [73]. The survival rate was reported to significantly drop in both breast and colon cancers as a result of MMP-9 overexpression [74]. In lung cancer, vascular endothelial growth factor (VEGF) induced the expression of MMP-9 and subsequently enhanced metastasis [75]. The design and synthesis of MMP-9 inhibitors is a potentially attractive research area. Unfortunately, to date, there is no effective MMP-9 inhibitor that successfully passed the clinical trials and gained FDA approval.

## 4. Computational Approaches and Synthesis of MMP-9 Inhibitors

The elevated expression of MMP-9 with various pathological conditions, including cancer, highlights the importance of designing, developing, and evaluating new MMP-9 inhibitors [76]. The comprehensive method of drug discovery involves chemical biology and computational drug design approaches. Chemical biology is important to elucidate the biological function of the target enzymes and the mechanism of their inhibition. On the other hand, computational approaches are crucial tools in drug design and development [77]. The two important types of computational approaches are structure-based drug design (SBDD) and ligand-based drug design (LBDD). In the case of MMP-9, SBDD methods are specialized to analyze the 3-dimensional structural information of the enzyme to identify the key structural features and significant interactions responsible for its biological activity. On the other hand, LBDD methods elucidate the 3-dimensional arrangements of structural commonalities between ligands and the targeted enzyme that are required for biological activity [78]. The complementary effect of those approaches has a powerful impact on rational drug design and development.

Mondal S. et al. [76] reviewed all the synthesized MMP-9 inhibitors with high activity and/or selectivity. Those inhibitors were tabulated with their selectivity, IC_50_ values, and chemical structures (Table S1). This study extensively explored the structural features of MMP-9 inhibitors [76].

In recent years, novel MMP-9 inhibitors have been designed, developed, and biologically investigated (Table 1). Hariono M. et al. [79] developed selective MMP-9 inhibitors bearing an aryl-amide linked to a heterocyclic ring toward the hemopexin domain of the enzyme. The mechanism of inhibition was investigated via FRET-based assay and gelatin zymography. In addition, an MTT assay was performed to assess the cytotoxicity of the inhibitors. Compounds **1**, **2**, and **3** were found to be the most potent inhibitors compared to the other synthesized aryl-amide derivatives. Indeed, those compounds demonstrated significant inhibition in the breast cancer cell line (4T1) with EC_50_ of 139 μM, 125 μM, and 132 μM, respectively [79].

Ayoup et al. [80] used a combinatorial approach to study different non-hydroxamate bis-amide scaffolds. They studied the potential of MMP-9/AKT inhibitors that are endowed with caspase 3/7 activation potential. They merged the pharmacophoric features of the lead compounds (P-nitrophenyl isonitrile, acids, amines, and aldehydes) via the Ugi reaction. It was found that compounds **4**, **5**, and **6** were the most active, selective, and safe derivates with single-digit nM IC_50_ against three types of cancer cell lines (MCF-7, NFS-60, and HepG-2) (Table 1). The compounds revealed a great selectivity to MMP-9 over MMP-1, -2, and -13 with a great caspase 3/7 activation potential (>51%). According to flow cytometric and metastasis analysis, the investigated compounds induced more than 49% apoptosis and up to 97% inhibition of cell migration in the examined cancer cell lines. According to drug-likeness metrics, compounds **4** and **6** were found to be drug-like candidates [80].

The halting of colorectal cancer was examined with a series of novel quinoxaline-based dual MMP-9/MAO-A inhibitors. The most important pharmacophoric features of MMP-9 and MAO-A inhibitors were merged and employed to yield a rationale scaffold design. Normal colon cells were used initially to assess the safety profiles of all derivatives, and then anticancer activity was assessed against HCT116 cells overexpressing MMP-9 and MAO-A. Among the evaluated series, compound **7** was found to be the most potent and selective dual MMP-9/MAO-A inhibitor (IC_50_ = 7.403 ± 0.201 nM) [81]. According to Haiba et al. [82], the s-triazine-based dendrimeric scaffold was developed to target tumor cells. This scaffold was supplemented with special pharmacophoric features that inhibit MMP-9 and halt cancer progression. Zinc-binding groups were utilized to design a rationale scaffold, including hydrazine branching chains, carboxylic acid, and hydroxamic acid, to obtain potential MMP-9 inhibitors. The MTT assay was used to assess the cytotoxicity effect of the synthesized series in both normal cells (Wi-38) and cancer cells (MDA-MB 231 and Caco-2). Compound **8** was the most potent inhibitor with high selectivity against MMP-9 in MDA-MB231 (IC_50_ = 3.8 ± 0.7 nM) and Caco-2(IC_50_ = 3.3 ± 0.5 nM). Molecular docking showed that the carboxylic acid group chelates the Zn active site of MMP-9 and forms a hydrogen bond with Tyr 423 in the ligand backbone [82].

Furthermore, some novel MMP-9 inhibitors revealed efficient docking scores with the targeted enzyme (Table 2). Kalva S. et al. [83] developed a pharmacophoric model to identify a new gelatinase inhibitor by using two highly active and selective MMP-9 inhibitors (PDB IDs: 2OVX and 2OW1). The E-pharmacophore model was composed of a hydrogen bond acceptor (HBA), hydrogen bond donor (HBD), and aromatic ring (RA). Model reliability was assessed by using the Guner–Henry (GH) scoring method and resulted in a 0.774 score, which signifies the ideal binding model for an MMP-9 inhibitor. The screening of 2,838,166 chemical structures was performed. Hierarchical clustering resulted in 33 groups according to their diversity. Furthermore, cross-docking studies of 33 molecules were performed on five MMPs, including MMP-1, -2, -8, -9, and -13. The highest docking scores with MMP-9 were shown only with two compounds, including compound **9** (−8.59 kcal mol^−1^) and compound **10** (−8.27 kcal mol^−1^). Molecular mechanics with generalized Born surface area (MM-GBSA) calculations revealed that the van der Waals interactions were the most crucial features behind high MMP-9 selectivity. The stability of ligands binding to the active site was assisted through molecular dynamics studies. It showed that compound **9** has a more stable binding than compound **10**. Consequently, compound **9** was a good lead compound that can be used to design and develop novel MMP-9 inhibitors [83].

**Figure 2:**
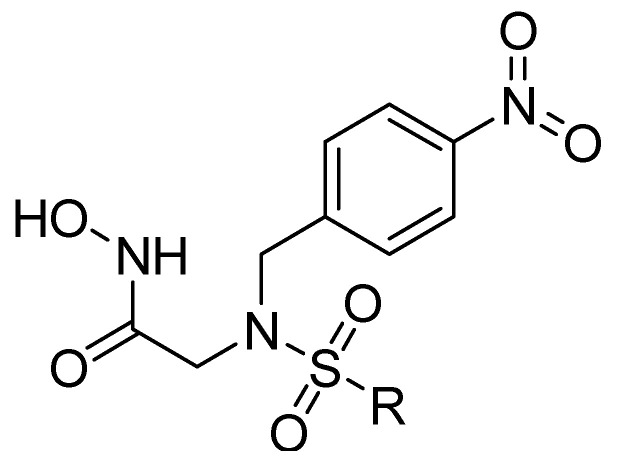
Scaffold of hydroximate derivatives. This figure was produced using ChemDraw version 7.

In addition, other known inhibitors have been used to determine a pharmacophore model, including NFH, Batimastat, Marimastat, Prinomastat, CGS-27023A, and Ro32-3555. The resulting model was composed of two hydrogen-bond acceptors (HBA), one hydrogen-bond donor (HBD), and one hydrophobic feature (HY). The assessment of the structure–activity relationship revealed that all the included inhibitors perfectly fitted the designed pharmacophore, except for CGS-27023A and Ro32-3555. These two compounds cannot fit properly with the second HBA. Those results emphasize the importance of the presence of HBA, HBD, and HY pharmacophore points in the model to ensure the potential affinity and biological activity of MMP-9 inhibitors [84]. Moreover, a 3D pharmacophore model was developed from a set of sixty-four β-N-biaryl ether sulfonamide hydroxamate derivatives as potent MMP-9 inhibitors. The best model was statistically significant and robust [85]. Compounds **11** and **12** presented higher predicted IC_50_ values of 9.94 nM and 9.88 nM, respectively. Additionally, Rathee et al., developed a pharmacophore model and 3D-QSAR model to predict the MMP-9 inhibitory activity of hydroximate derivatives (Figure 2). The five-point (AAARR) pharmacophoric model was shown with three hydrogen-bond acceptors (HBA) and two aromatic rings (RA). After hypothesis validation, the best 3D-QSAR model was determined. The best model revealed that the substitution at C3, 4, and 5 with electron withdrawing, ionic, or/and hydrophobic groups is critical in MMP-9 activity. On the other hand, no significant effect on MMP-9 activity was revealed upon the substation in C2 and 6. This QSAR model was found to be a beneficial way to predict the structure–activity relationship of the hydroxamate derivative in order to design and develop a new gelatinase inhibitor [86].

**Figure 3:**
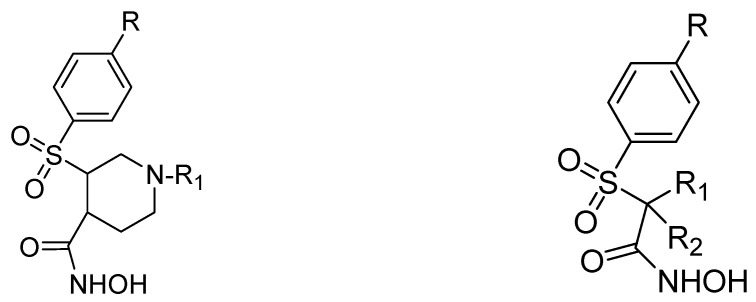
Scaffold of N-hydroxy-α-phenylsulfonyl acetamide (HPSAs) derivatives. This figure was produced using ChemDraw version 7.

Another study was conducted on 80 N-hydroxy-α-phenylsulfonyl acetamide (HPSAs) derivatives to reveal the essential structural feature required for high binding affinity (Figure 3). Two QSAR models have been developed with robust statistical results. The first model is composed of two hydrogen-bond acceptors (HBA), two hydrogen-bond donors (HBD), and one aromatic ring (RA). The second model is composed of three hydrogen-bond acceptors (HBA), one positive ionic (P), and one aromatic ring (RA). These results could be highly beneficial in the prediction of the structure–activity relationship of HPSA derivatives as MMP-9 inhibitors [87].

Furthermore, three ligand-based pharmacophore models have been developed based on known MMP-9 inhibitors. The best model is composed of two hydrogen-bond acceptors (HBA), one hydrogen-bond donor (HBD), one hydrophobic group (HY), and one aromatic ring (AR). Through high-throughput virtual screening and molecular docking approaches, many compounds exhibited a high binding affinity and selectivity to MMP-9 enzyme, including compounds **13**, **14**, **15**, **16**, **17**, **18**, **19**, **20**, and **21** [88]. In 2021, an additional study was conducted with 67 MMP-9 inhibitors having *P*IC_50_ values ranging from 5.221 nM to 9.000 nM. Sanapalli et al., designed and validated a robust five-point 3D-QSAR model that contains two hydrogen-bond donors (HBD), one hydrophobic region (HY), and two aromatic rings (RA). Through virtual screening and molecular docking approaches, two compounds were identified as selective MMP-9 inhibitors, including compounds **22** and **23**, due to their significant interactions with the active residues [89].

## 5. Natural MMP-9 Inhibitors

**Figure 4:**
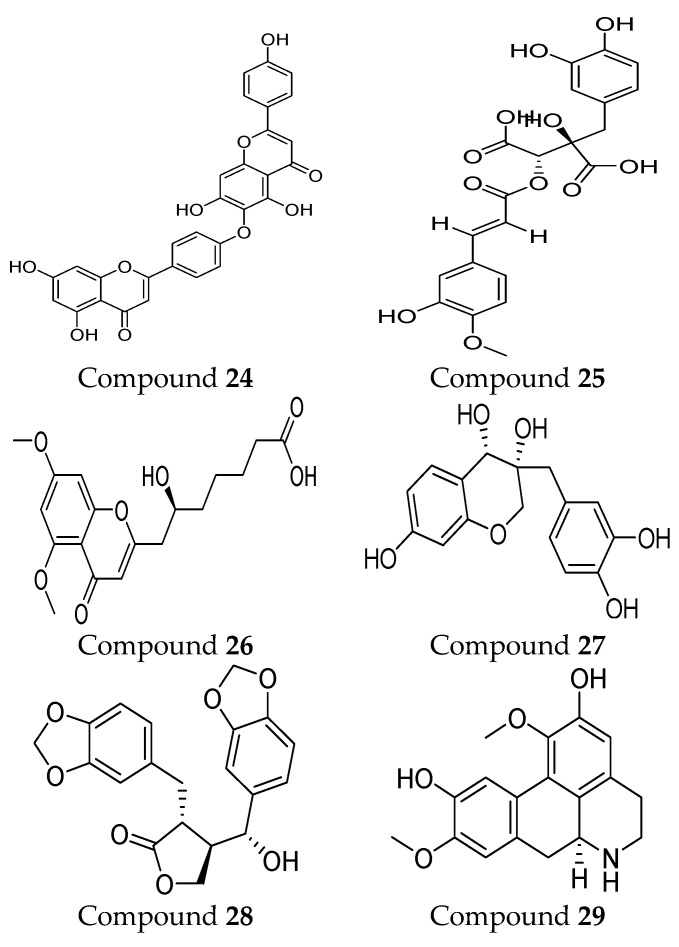

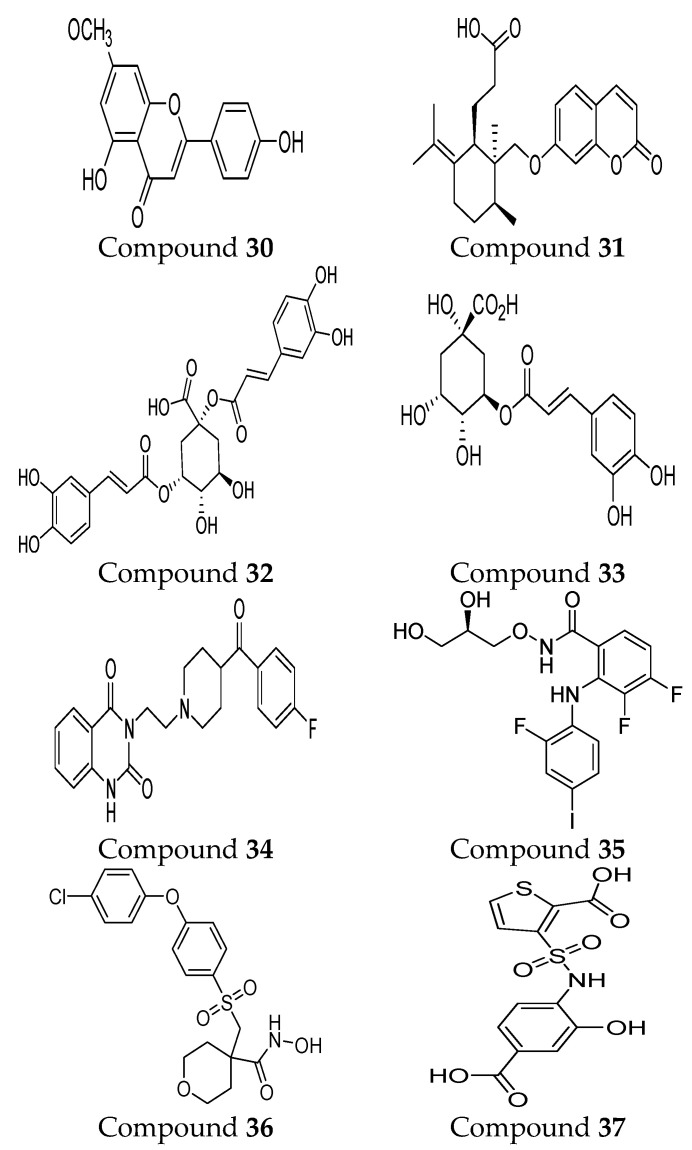
Molecular structures of natural MMP-9 inhibitors. All chemical structures were produced using ChemDraw version 7.

In recent years, interest in natural MMP-9 inhibitors has markedly increased. For instance, some natural inhibitors exhibited a potential MMP-9 inhibitory activity and were discovered through various computational approaches (Figure 4). Kalva S. et al. [90] developed a ligand-based pharmacophore model from different classes of natural MMP-9 inhibitors [90]. The Güner–Henry (GH) scoring method is used to validate the generated pharmacophoric model. It consists of three hydrogen-bond acceptors (HBA) and two aromatic ring regions (RA). The screening of natural compounds was conducted on this model to identify novel MMP-9 inhibitors. Through molecular docking and dynamic simulation studies, compound **24** (from *Juniperus communis*) demonstrated high binding free energy (−26.54 KJ/mol) when compared with the known inhibitors of MMP-9. The results of the MTT assay on the human breast cancer MCF-7 cell line (IC_50_ = 43.08 µM) proved the effectiveness of the developed pharmacophoric model in MMP-9 inhibitor design [90]. Moreover, another pharmacophoric model was developed based on the experimental binding manner of natural products with MMP-9. This model consists of six structural features, including two hydrogen-bond acceptors (HBA), one hydrogen-bond donor (HBD), one ring aromatic region (RA), and two hydrophobic (HY) groups. The hydrophobic feature was incredibly important because they recognize and bind to the S1′ pocket, which is essential for MMP-9 selectivity. Compound **25** was recognized as a potent MMP-9 inhibitor. An in vitro assay was conducted on the quenched fluorogenic substrate for MMPs (Mca-PLGL-Dpa-AR-NH2) and proved the reliability of the developed model. This revealed a strong inhibitory activity of compound **25** against MMP-9 with IC_50_ = 13.4 µM [91].

Furthermore, the 3D-QSAR pharmacophore model development of natural compounds was conducted. This model contains two hydrogen-bond acceptors (HBA), one hydrophobic (HY), and one ring aromatic (RA). The results of this model are consistent with a previous study that emphasized the importance of the HY and RA group for the high binding affinity and selectivity due to the interaction with the S1′ pocket [91]. Compound **26** revealed a potential inhibitory activity toward the MMP-9 enzyme through virtual screening and molecular docking approaches. In vitro assessment yielded a strong inhibitory activity in MMP-9 (IC_50_ = 26.94 µM). Additional methods were used to evaluate model reliability through hybrid quantum mechanics/molecular mechanics (QM/MM) calculation and molecular dynamics simulation. Satisfactory results were reported due to powerful binding with the MMP-9 active site through various types of interactions [92].

Another computational study was conducted to develop a targeted therapy for clear cell renal cell carcinoma (CCRCC) through the screening of ideal natural compounds. Discovery Studio 4.5 is utilized to compare the MMP-9 binding affinity of the standard drug (solasodine) with the ligands. Among the top 20 ligands, compound **27** and compound **28** showed undefined blood–brain barrier levels, lower aqueous solubility, hepatotoxicity, and carcinogenicity compared to the standard compound. According to the CHARMM force field, the CDOCKER energies of binging were calculated and revealed that compound **27** (−50.817 kcal/mol) and compound **28** (−51.7422 kcal/mol) bind more firmly than the standard compound (−23.1805 kcal/mol). The pharmacophore model of compound **27** includes 33 structural features (14 HBA, 13 HBD, 2 HY, and 4 AR). Compound **28** showed 23 pharmacophore features (12 HBA, 5 HBD, 2 HY, and 4 AR). On the other hand, the standard compounds revealed the least pharmacophoric features composed of 18 points (7 HBA, 6 HBD, 4 HY, and 1 P). The in vitro effectiveness of those compounds was proved by using cell-counting kit-8, colony-forming, and scratch assays. These compounds can present valuable insights into CCRCC-targeted therapy design and development [93].

In 2022, two MMP-9 inhibitors were discovered, including compound **29** and compound **30**, through a quantum mechanical fragment molecular orbital (FMO)-based virtual screening method. Pharmacophore model development and quantitative binding-affinity measurements were conducted. The inhibitors showed a dissociation constant (K_D_) of 21.6 µM and 0.614 μM, respectively. These results indicated the important role of novel FMO-based methods in identifying new natural MMP-9 inhibitors [94].

**Figure 5:**
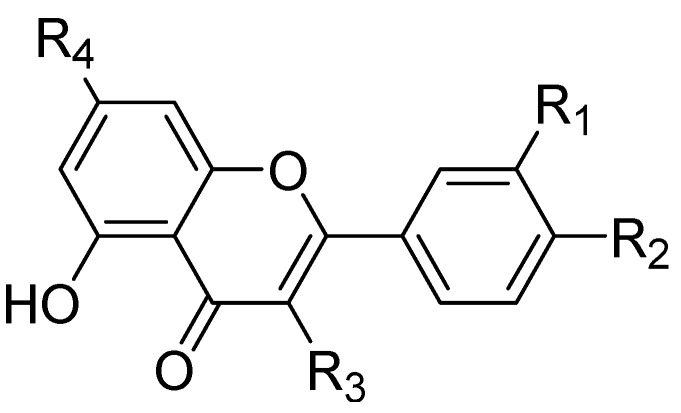
Molecular structure of the studied flavonoids. This figure was produced using ChemDraw version 7.

Zhou Z.G. et al. [95] studied the mechanism of MMP-9 inhibition by a flavonoid inhibitor through different approaches, including molecular docking, hybrid quantum mechanical and molecular mechanical (QM/MM) calculations, and molecular dynamics simulations (Figure 5). Experimentally proven flavonoids were involved in the study, including primuletin, chrysin, apigenin, luteolin, and quercetin. A good linear correlation was shown between the calculated binding free energies of the five flavonoids with the previous experimental log (EC_50_) values. Various hydrogen bond networks in addition to Zn-O coordination bonds were revealed from the developed binding modes of the five studied flavonoids. The obvious consistency between these results with previously conducted experimental studies revealed the potential effectiveness and reliability of the force field parameters in the investigation process of flavonoid–MMP-9 interactions [96,97,98].

Furthermore, the inhibitory activity of compound **31** in MMP-9 was investigated through several approaches, including absorption spectroscopy, FT-IR, molecular docking, and molecular dynamics (MDs) simulation procedures. The results revealed the potential binding of this compound to MMP-9 in the hydrophobic cavity’s (Ala 189, Leu187, Ala 19, Tyr245, Pro246, and Met247) nearness to the Zn atom. A molecular dynamics simulation study showed that it was bound to the MMP-9 with little modifications of its tertiary structure and without any influence on the secondary structure of the protein. Additionally, they revealed the potential role of hydrogen bond interactions between galbenic acid and MMP-9 [99].

Malekipour M.H. et al. [100] evaluated the binding affinity of cinnamic acid derivatives on the MMP-9 active site. A salient binding affinity was revealed with three cinnamic acid derivatives (compound **32**, compound **33**, and compound **34**) (Δ*G*_binding_ < −10 kcal/mol). Compound **32** and compound **33** revealed potential inhibitory activities at the picomolar scale (17.37 pM and 557.56 pM, respectively). Additionally, stable docked poses were represented by both inhibitors in the ps simulation. Recently, new inhibitors were discovered targeting catalytic Zn^2+^ ion and S1 pocket of MMP-9 via deep learning-based method followed by molecular docking and molecular dynamics simulation approach. Four compounds demonstrated a high stability and selectivity, including compounds **34**, **35**, **36**, and **37**. The radius of gyration and MD simulation study showed the compactness of the proteins and the great stability of all protein–ligand complexes. Indeed, the stable interactions of those compounds with the catalytic domain were correlated to the continuous contribution of hydrogen bonding interactions. Interestingly, those inhibitors exhibited higher and significantly better interaction energies with MMP-9 than the reference compound used in the study [101].

## 6. MMP-9 Inhibitors in Clinical Trials

**Figure 6:**
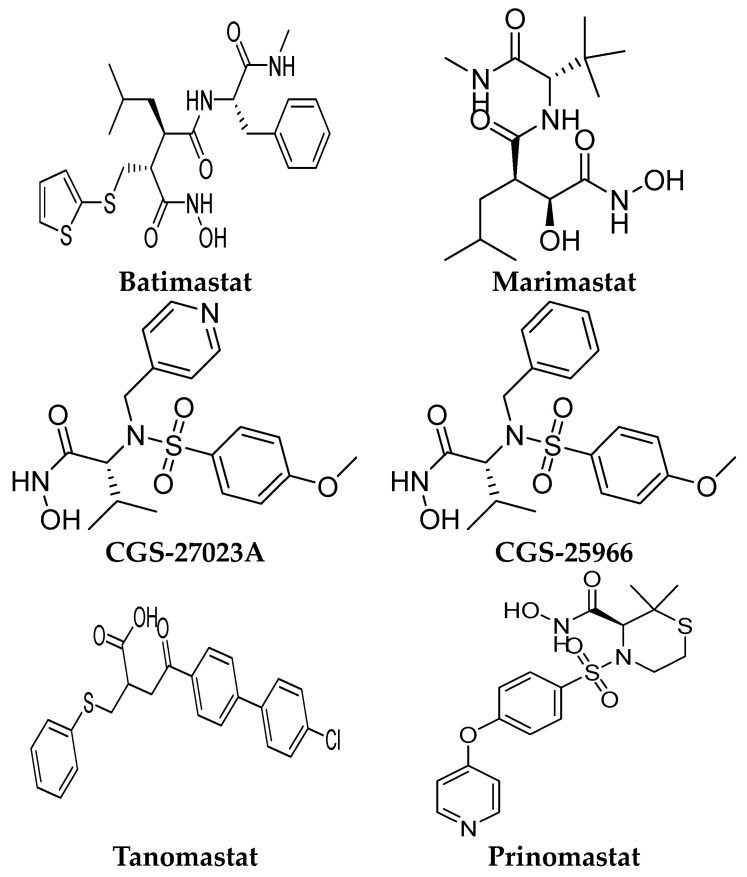
Molecular structures of the clinically investigated MMP-9 inhibitors. All chemical structures were produced using ChemDraw version 7.

Several MMP-9 inhibitors were enrolled in the clinical investigation against different types of cancer (Figure 6). Those inhibitors were investigated either alone or in combination with other well-characterized anticancer drugs.

### 6.1. Batimastat (BB-94)

Batimastat is a peptidomimetic compound with a hydroxamic acid moiety. It is the first MMP inhibitor to be evaluated in clinical trials [102]. It induced a significant antiproliferative activity in various preclinical cancer models, including orthotopic tumor xenografts model [103], metastasis models [104,105,106], and ovarian cancer models [107]. Early stage tumors were found to be more sensitive to batimastat than tumors in later stages. The drug exhibited a synergistic antiproliferative effect with docetaxel and captopril [108]. In addition, the growth-suppressive activity of cisplatin was potentiated by batimastat [109]. In 1991, the first clinical trial of batimastat was conducted. The drug was used as a capsule formulation, but it revealed poor bioavailability [110]. In 1993, a phase I clinical trial was conducted by using an intra-peritoneal formulation of batimastat [111]. Twenty-three patients with malignant ascites were recruited for the study. The drug was well-tolerated; no serious adverse effects and no toxicities were seen. It was found to be effective in symptoms of palliation, especially malignant effusion. Early signs of batimastat efficacy were revealed with a reduced need for paracentesis for several patients. Sixteen out of twenty-three patients did not require re-drainage within 28 days from starting the treatment [111]. In 1999, another phase I study of batimastat was conducted on eighteen patients with positive malignant pleural effusions. It was found to be well-tolerated. Toxic effects were reported, including low-grade fever and the reversible elevation of liver enzymes. Those effects were found to be non-related to the batimastat dose or plasma level. Sixteen patients significantly required fewer pleural aspiration, and seven patients required no further aspiration after the treatment. In addition, improvement in dyspnea scores and significant enhancement in exercise tolerance was reported with batimastat [112]. This drug was not continued for the next clinical stages due to poor solubility in oral preparations, poor selectivity, produced mild systemic toxicity, marked abdominal pain, and plural infusion [112].

### 6.2. Marimastat (BB-2516)

Marimastat is a synthetic low-molecular-weight inhibitor containing a collagen-mimicking hydroxamate structure. In contrast to batimastat, it is available in oral formulations with a good bioavailability of 20–50% due to the presence of hydroxyl and tert-butyl groups [113]. Through pre-clinical studies, the effectiveness of marimastat was proved against breast and lung metastasis models [113]. In 1998, a phase I study was conducted to evaluate marimastat safety and pharmacokinetic properties in twelve patients with advanced lung cancer. Toxic effects were reported at doses of 100–500 mg/kg per day, including gastrointestinal adverse reactions, hemorrhage, fibrosis, and necrosis at the periarticular ankle [114]. Subsequently, several phase I–II clinical trials of marimastat were conducted either alone or in combination with other chemotherapy [114,115,116,117,118]. Clinical trials of marimbists were conducted on diverse types of cancer, including breast, lung, prostate, ovarian, pancreas, melanoma, and colon cancer. The main toxic effect of this compound was found to be dose-limiting inflammatory polyarthritis, which was reported mostly during the first month of treatment [114]. Interestingly, marimastat entered phase III clinical trials alone or in combination with patients with several types of cancer. A significant improvement was shown in gastric cancer patients [119]. In addition, a randomized double-blind placebo-controlled trial of marimastat was conducted with lung cancer patients. No significant difference was found in the median survivals for marimastat and placebo patients (9.3 months and 9.7 months, respectively) (*p* = 0.90). This compound did not reveal a significant improvement when used after induction therapy for lung cancer [117].

### 6.3. CGS-27023A (MMI-270)

It is a small-molecule sulphonamide derivative MMP inhibitor. This compound is a non-peptidomimetic inhibitor that suppresses tumor growth as proved through preclinical studies [120]. It is a broad-spectrum inhibitor and is available in an oral formulation. This compound was terminated in phase I clinical trial studies with non-small-cell lung carcinoma due to the reported side effects, mainly joint and muscle pain [37].

### 6.4. CGS-25966

It is a broad-spectrum hydroxamic-acid derivative. This compound was terminated due to pharmacokinetic issues, including poor solubility and low oral bioavailability [40]. In addition, severe side effects including musculoskeletal syndrome (joint stiffness, pain, tendinitis, and inflammation) were reported with this treatment [40,121].

### 6.5. Tanomastat (BAY12-9566)

It is an orally bioavailable biphenyl compound that contains a carboxylate group as a catalytic zinc ion chelator. In vitro, this compound yielded tumor growth inhibition against the breast cancer orthotopic model [122]. According to phase I studies, the recommended dose for phase II clinical trials of BAY12-9566 was reported to be 800 mg b.i.d after oral administration [123,124]. In addition, another phase I study of BAY 12-9566 in combination with etoposide and carboplatin was conducted in patients with advanced cancer. The combination of BAY 12-9566 with etoposide was safe and well-tolerated, while the combination of BAY 12-9566 with etoposide and carboplatin resulted in significant hematological toxicity [125]. In a phase III randomized trial, this compound was well-tolerated in patients with ovarian cancer but exhibited non-significant improvement in progression-free survival (PFS) or median overall survival (OS) [126]. The inferior antitumor activity of BAY 12-9566 versus gemcitabine was revealed through a phase III clinical trial in patients with advanced pancreatic adenocarcinoma [127].

### 6.6. Prinomastat (AG-3340)

It is a nonpeptidic collagen-mimicking inhibitor, available in oral and intraperitoneal dosage forms. In the phase III clinical trial, prinomastat did not improve the activity of chemotherapy (gemcitabine and cisplatin) in patients with non-small-cell lung cancer (NSCLC) [128]. Furthermore, venous thromboembolism (VTE) was reported as severe toxicity associated with prinomastat when used in combination with other chemotherapy (gemcitabine/cisplatin or paclitaxel/carboplatin) in NSCLC [129]. Additionally, moderate to severe musculoskeletal toxicity was reported in the phase II study when prinomastat was used preoperatively in patients with esophageal adenocarcinoma [130]. The development of this inhibitor was terminated due to a lack of efficacy as an antitumor in addition to the high toxicity reported in the clinical trials.

## 7. Conclusions and Perspective

The function of metalloproteinase potentially affects the pathophysiological functions of the human body. Therefore, the blockade of this enzyme family may produce some systemic effects accompanied by the inhibition of cancer cell invasion and metastasis. Consequently, research on new anticancer drugs with high selectivity and safety profiles is needed. MMP-9 is a metalloproteinase that has an important role in ECM remodeling, angiogenesis, metastasis, and cancer progression. Hence, MMP-9 inhibitors have become a potential target for anticancer drug development. According to previously conducted structural and computational studies, several MMP-9 inhibitors have been developed with different scaffolds. Most of the reported inhibitors revealed non-selective binding and little efficacy due to high sequence homology with other MMPs. Hence, none of these inhibitors gained FDA approval for cancer treatment. Therefore, the design, synthesis, and development of selective MMP-9 inhibitors will be highly useful in anticancer therapy and will minimize off-target adverse effects.

A comprehensive method of drug discovery, which includes chemical biology and computational drug design approaches, should be employed to shed light on the multiple aspects of developing selective MMP-9 inhibitors. Among the computational approaches, molecular modeling aspects may be helpful to gain a great acceptance for reducing time and effort. For MMP-9 selectivity, amino acid residues that form the S1′ pocket play a major role. Additionally, the type of hydrophobic aryl group in the ligand structure that is directed to fit the S1′ pocket also has a potential effect on the selectivity of MMP-9 inhibitors. Depending on these specific features and techniques, novel MMP-9 inhibitors with potential anticancer activity may be designed.

## Figures and Tables

**Table 1:** The chemical structures of synthetic MMP-9 inhibitors with their IC_50_/EC_50_ values. All chemical structures were produced using ChemDraw version 7.

Inhibitor	Cell Line	IC_50_/EC_50_	Chemical Structure
Compound **1** [79]	4T1 cells	EC_50_ = 139 µM	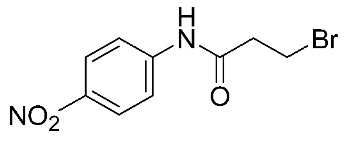
Compound **2** [79]	4T1 cells	EC_50_ = 125 µM	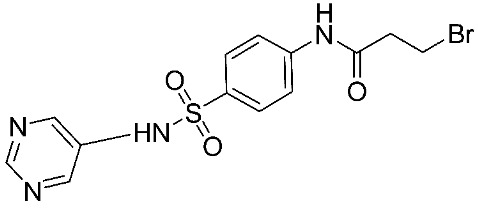
Compound **3** [79]	4T1 cells	EC_50_ = 132 µM	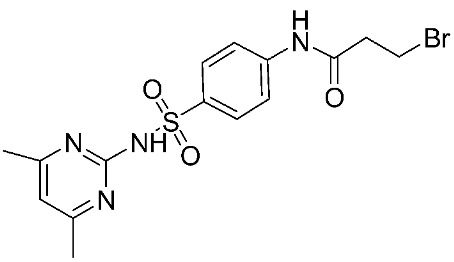
Compound **4** [80]	MCF-7	IC_50_ = 6.9 ± 0.5 nM	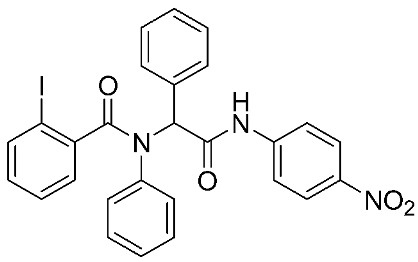
	NFS-60	IC_50_ = 5.5 ± 0.6 nM	
	HepG-2	IC_50_ = 3.1 ± 0.4 nM	
Compound **5** [80]	MCF-7	IC_50_ = 3.6 ± 0.4 nM	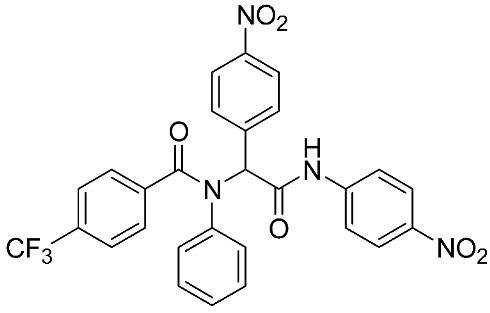
	NFS-60	IC_50_ = 3.61 ± 0.1 nM	
	HepG-2	IC_50_ = 2.2 ± 0.1 nM	
Compound **6** [80]	MCF-7	IC_50_ = 6.6 ± 0.7 nM	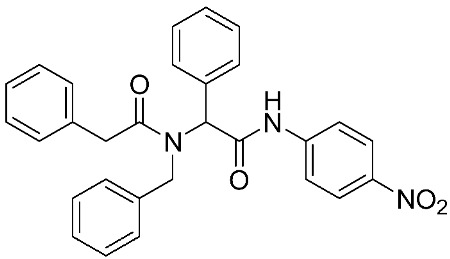
	NFS-60	IC_50_ = 5.8 ± 0.2 nM	
	HepG-2	IC_50_ = 5.7 ± 1 nM	
Compound **7** [81]	HCT116	IC_50_ = 7.403 ± 0.201 nM	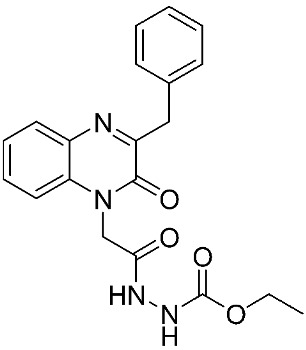
Compound **8** [82]	MDA-MB231	IC_50_ = 3.8 ± 0.7 nM	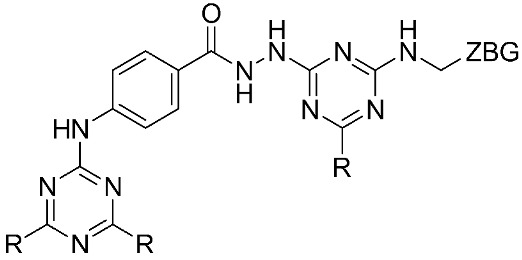
	Caco-2	IC_50_ = 3.3 ± 0.5 nM	

**Table 2:** The chemical structures of synthetic MMP-9 inhibitors with their docking score values. All chemical structures were produced using ChemDraw version 7.

Inhibitor	Docking Score (kcal mol^−1^)	Chemical Structure
Compound **9** [83]	−8.59	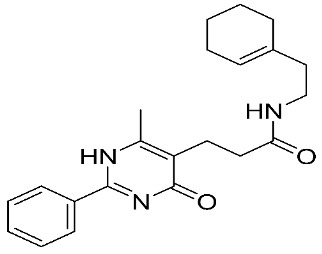
Compound **10** [83]	−8.27	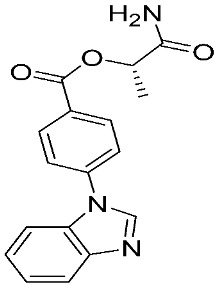
Compound **11** [85]	−42.28	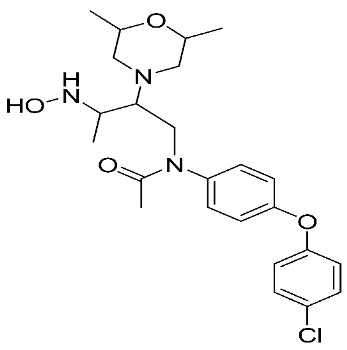
Compound **12** [85]	−40.53	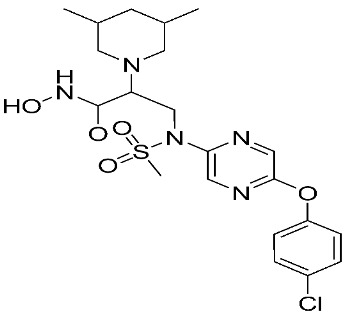
Compound **13** [88]	−10.322	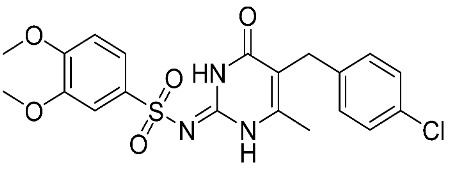
Compound **14** [88]	−10.03	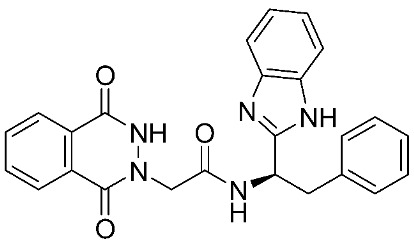
Compound **15** [88]	−9.983	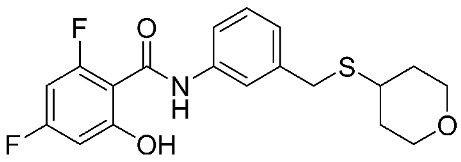
Compound **16** [88]	−9.783	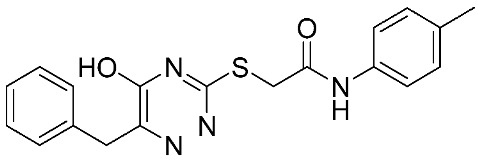
Compound **17** [88]	−9.434	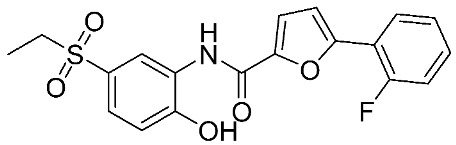
Compound **18** [88]	−9.406	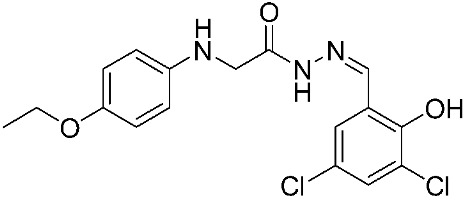
Compound **19** [88]	−9.251	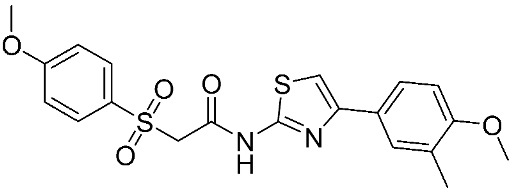
Compound **20** [88]	−9.06	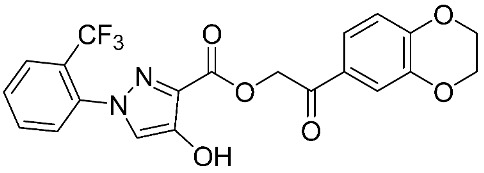
Compound **21** [88]	−9.029	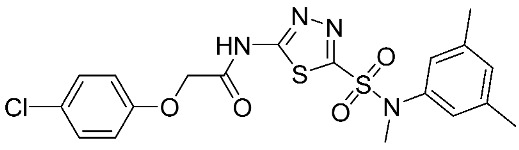
Compound **22** [89]	−10.967	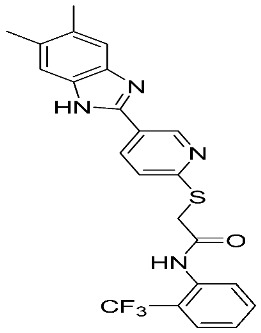
Compound **23** [89]	−9.948	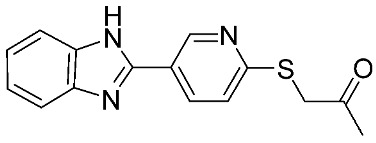

## Data Availability

Data supporting the findings of this study are available within the article.

## Supplementary Materials

The following supporting information can be downloaded at: https://www.mdpi.com/article/10.3390/ijms241512133/s1. References [24,26,80,131,132,133,134,135,136,137,138,139,140,141,142,143,144,145,146,147,148,149,150,151,152,153,154,155,156,157,158,159,160,161,162,163,164,165,166] are cited in the Supplementary Materials.
