# Supplementary material for: Novel Matrix Metalloproteinase-9 (MMP-9) Inhibitors in Cancer Treatment
Source: Int J Mol Sci. 2023 Jul 28;24(15):12133. doi: 10.3390/ijms241512133 (PMC10418771; doi:10.3390/ijms241512133)
Supplement: Supplementary file 1 [file ijms-24-12133-s001.zip › ijms-2479745-supplementary.pdf]

Table S1. The chemical structures of synthetic MMP-9 inhibitors with their IC_{50} values. All chemical structures were produced using ChemDraw version 7.

Inhibitor	Selectivity	MMP-9 IC50	Structure
**Compound 1** [1]	Selective	0.2 nM	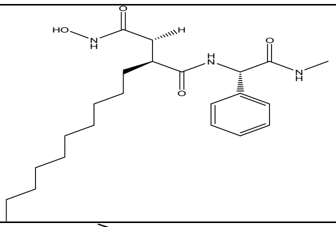
**Compound 2** [2]	Non-selective	0.2 nM	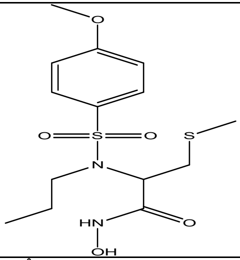
**Compound 3** [3]	Non-selective	5 nM	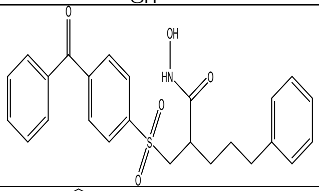
**Compound 4** [4]	Selective	0.01 nM	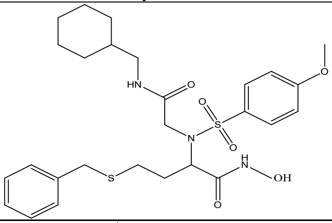
**Compound 5** [5]	Selective	0.9 nM	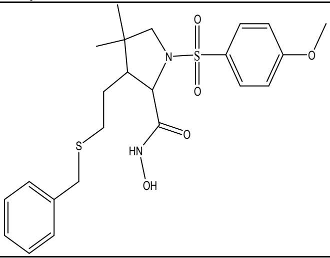
**Compound 6** [6]	Selective	0.3 nM	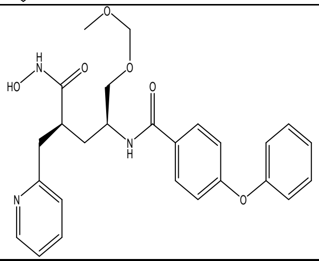

**Compound 7** [7]	Selective	0.52 nM	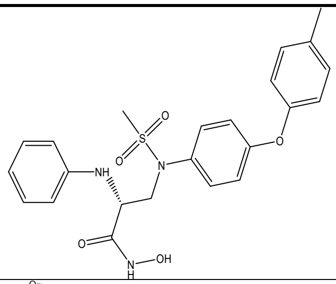
**Compound 8** [8]	Selective	0.80 nM	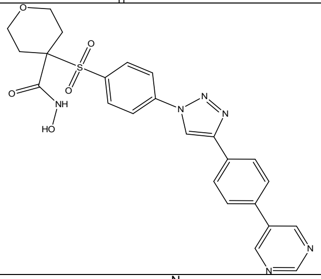
**Compound 9** [9]	Selective	0.54 nM	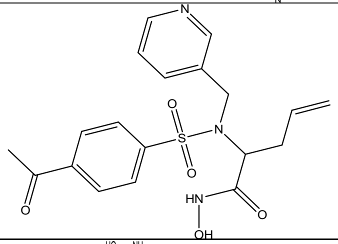
**Compound 10** [10]	Selective	0.43 nM	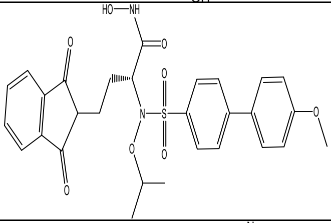
**Compound 11** [11]	Selective	1.06 nM	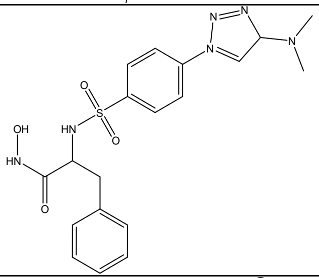
**Compound 12** [12]	Selective	3 nM	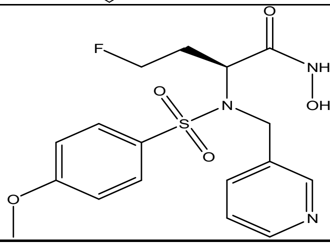

**Compound 13** [13]	Non-selective	4.4 nM	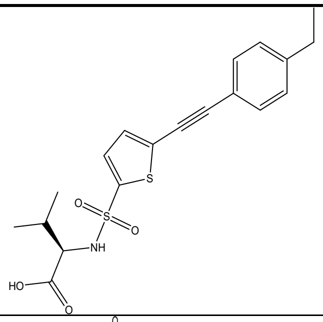
**Compound 14** [14]	Non-selective	0.5 nM	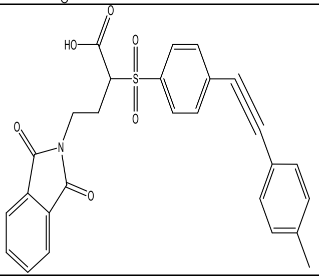
**Compound 15** [15]	Non-selective	1 nM	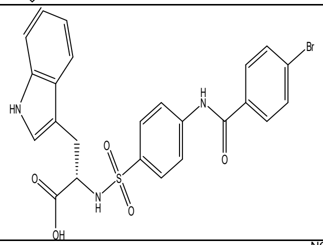
**Compound 16** [16]	Selective	4.83 μM	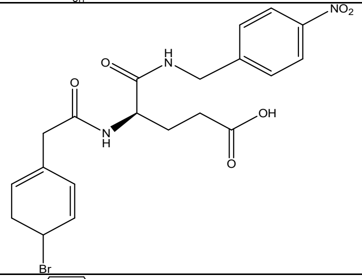
**Compound 17** [17]	Non-selective	492 nM	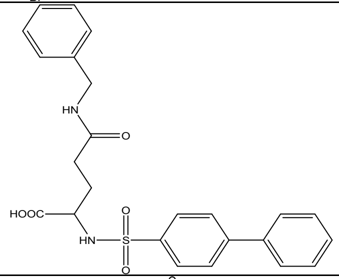
**Compound 18** [18]	Non-selective	4.8 μM	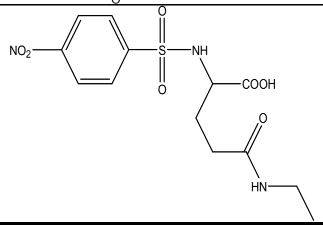

**Compound 19** [19]	Selective	4.49 nM	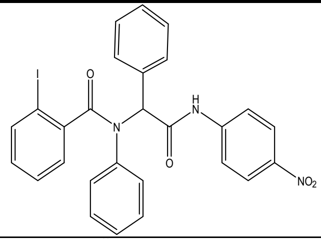
**Compound 20** [20]	Non-selective	36.4 nM	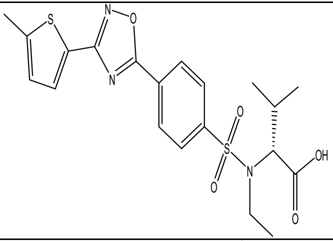
**Compound 21** [21]	Selective	0.092 nM	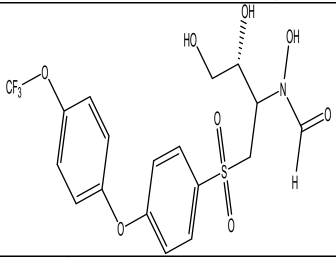
**Compound 22** [21]	Selective	0.34 nM	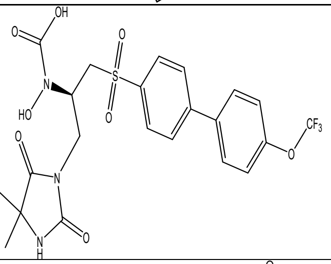
**Compound 23** [22]	Selective	1 nM	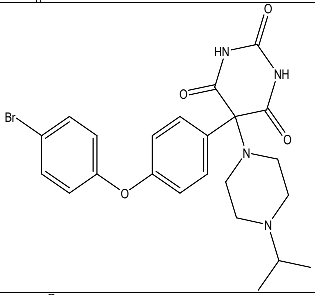
**Compound 24** [23]	Non-selective	1.1 nM	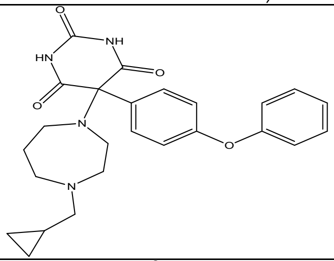
**Compound 25** [24]	Selective	9.1 nM	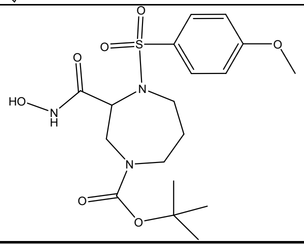

**Compound 26** [25]	Selective	14 nM	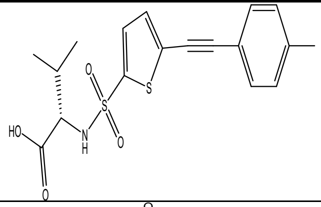
**Compound 27** [26]	Selective	32 nM	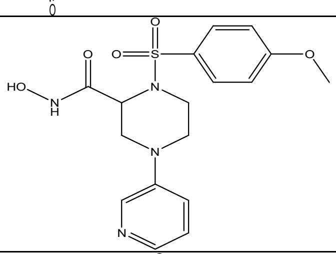
**Compound 28** [27]	Selective	6.8 nM	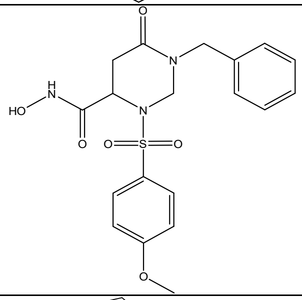
**Compound 29** [28]	Selective	1 nM	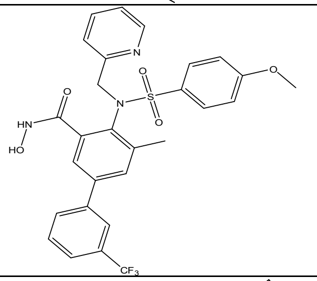
**Compound 30** [29]	Selective	187 nM	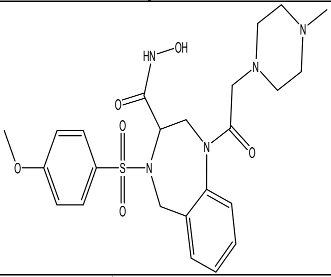
**Compound 31** [30]	Non-selective	9 nM	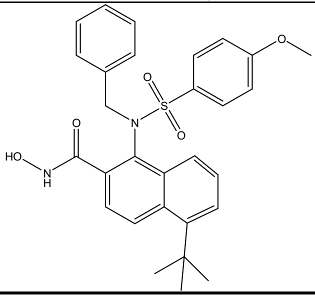

**Compound 32** [31]	Selective	3 nM	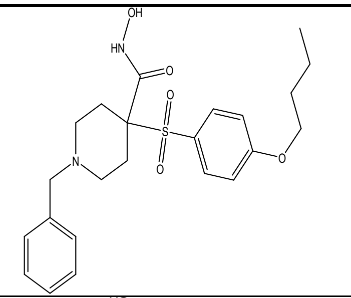
**Compound 33** [32]	Selective	13 nM	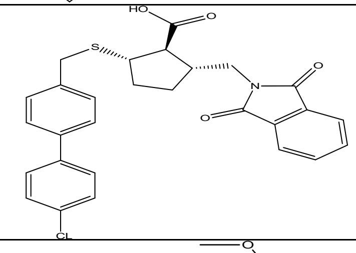
**Compound 34** [33]	Selective	0.87 nM	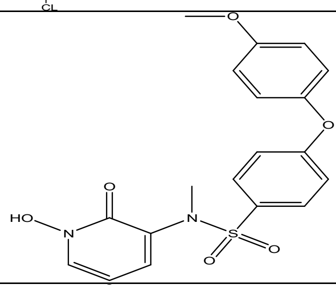
**Compound 35** [7]	Selective	0.89 nM	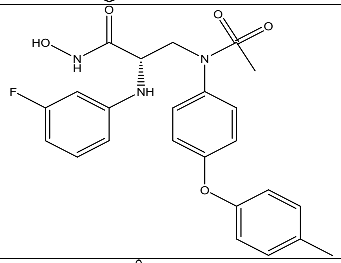
**Compound 36** [34]	Selective	410 nM	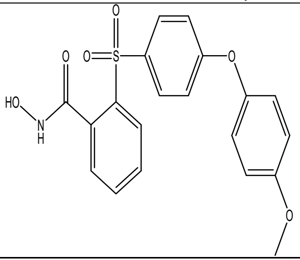
**Compound 37** [35]	Selective	0.1 nM	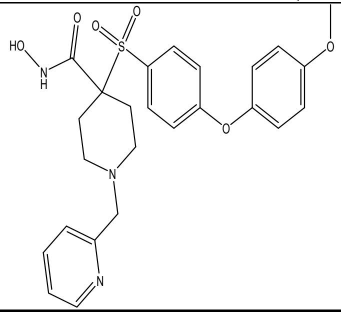

**Compound 38** [36]	Selective	0.22 nM	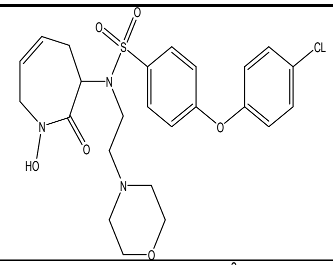
**Compound 39** [37]	Selective	2 nM	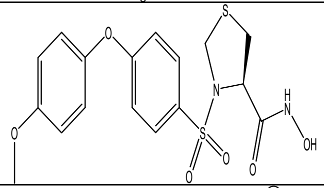
**Compound 40** [38]	Selective	2 nM	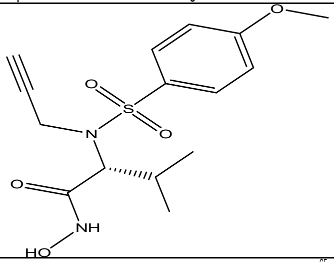
**Compound 41** [39]	Selective	29.6% inhibition at 20 micro-mol	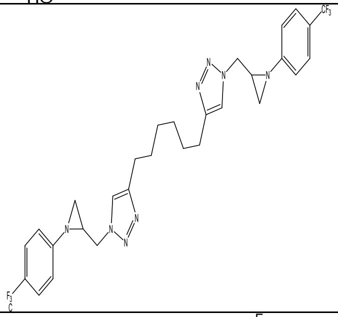
**Compound 42** [9]	Non-selective	3 nM	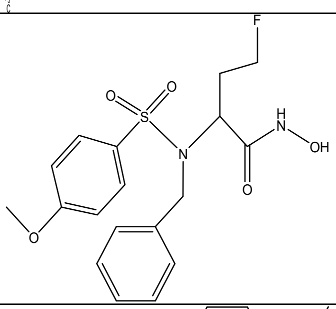
**Compound 43** [40]	Selective	440 nM	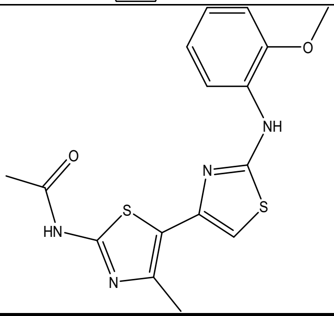

References:

1. Yamamoto, M.; Tsujishita, H.; Hori, N.; Ohishi, Y.; Inoue, S.; Ikeda, S.; Okada, Y. Inhibition of Membrane-Type 1 Matrix Metalloproteinase by Hydroxamate Inhibitors: An Examination of the Subsite Pocket. *J. Med. Chem.* **1998**, *41*, 1209–1217, https://doi.org/10.1021/jm970404a.
2. Hanessian, S.; Bouzbouz, S.; Boudon, A.; Tucker, G.C.; Peyroulan, D. Picking the S1, S1′ and S2′ pockets of matrix metalloproteinases. A niche for potent acyclic sulfonamide inhibitors. *Bioorganic Med. Chem. Lett.* **1999**, *9*, 1691–1696, https://doi.org/10.1016/s0960-894x(99)00259-0.
3. Baxter, A.D.; Bhogal, R.; Bird, J.; Keily, J.F.; Manallack, D.T.; Montana, J.G.; A Owen, D.; Pitt, W.R.; Watson, R.J.; E Wills, R. Arylsulphonyl hydroxamic acids: potent and selective matrix metalloproteinase inhibitors. *Bioorganic Med. Chem. Lett.* **2001**, *11*, 1465–1468, https://doi.org/10.1016/s0960-894x(01)00259-1.
4. Hanessian S, Moitessier N, Gauchet C, Viau MJJomc. N-Aryl sulfonyl homocysteine hydroxamate inhibitors of matrix metalloproteinases: further probing of the S1, S1 ′, and S2 ′pockets. 2001;44:3066-73 https://doi.org/10.1021/jm010097f.
5. Hanessian, S.; MacKay, D.B.; Moitessier, N. Design and Synthesis of Matrix Metalloproteinase Inhibitors Guided by Molecular Modeling. Picking the S_1Pocket Using Conformationally Constrained Inhibitors. *J. Med. Chem.* **2001**, *44*, 3074–3082, https://doi.org/10.1021/jm010096n.
6. Yamamoto, S.; Nakatani, S.; Ikura, M.; Sugiura, T.; Nishita, Y.; Itadani, S.; Ogawa, K.; Ohno, H.; Takahashi, K.; Nakai, H.; et al. Design and synthesis of an orally active matrix metalloproteinase inhibitor. *Bioorganic Med. Chem.* **2006**, *14*, 6383–6403, https://doi.org/10.1016/j.bmc.2006.05.040.
7. Yang S-M, Scannevin RH, Wang B, Burke SL, Huang Z, Karnachi P, et al. β-N-Biaryl ether sulfonamide hydroxamates as potent gelatinase inhibitors: Part 2. Optimization of α-amino substituents. 2008;18:1140-5.
8. Fabre, B.; Filipiak, K.; Zapico, J.M.; Díaz, N.; Carbajo, R.J.; Schott, A.K.; Martínez-Alcázar, M.P.; Suárez, D.; Pineda-Lucena, A.; Ramos, A.; et al. Progress towards water-soluble triazole-based selective MMP-2 inhibitors. *Org. Biomol. Chem.* **2013**, *11*, 6623–6641, https://doi.org/10.1039/c3ob41046c.
9. Behrends M, Wagner S, Kopka K, Schober O, Schaefers M, Kumbhar S, et al. New matrix metalloproteinase inhibitors based on γ-fluorinated α-aminocarboxylic and α-aminohydroxamic acids. 2015;23:3809-18.
10. Nuti, E.; Cantelmo, A.R.; Gallo, C.; Bruno, A.; Bassani, B.; Camodeca, C.; Tuccinardi, T.; Vera, L.; Orlandini, E.; Nencetti, S.; et al. *N-O*-Isopropyl Sulfonamido-Based Hydroxamates as Matrix Metalloproteinase Inhibitors: Hit Selection and in Vivo Antiangiogenic Activity. *J. Med. Chem.* **2015**, *58*, 7224–7240, https://doi.org/10.1021/acs.jmedchem.5b00367.
11. Zapico, J.M.; Puckowska, A.; Filipiak, K.; Coderch, C.; de Pascual-Teresa, B.; Ramos, A. Design and synthesis of potent hydroxamate inhibitors with increased selectivity within the gelatinase family. *Org. Biomol. Chem.* **2014**, *13*, 142–156, https://doi.org/10.1039/c4ob01516a.
12. Hugenberg V, Behrends M, Wagner S, Hermann S, Schäfers M, Kolb HC, et al. Synthesis, radiosynthesis, in vitro and first in vivo evaluation of a new matrix metalloproteinase inhibitor based on γ-fluorinated α-sulfonylaminohydroxamic acid. 2018;3:1-20, https://doi.org/10.1186/s41181-018-0045-0.
13. Kiyama, R.; Tamura, Y.; Watanabe, F.; Tsuzuki, H.; Ohtani, M.; Yodo, M. Homology Modeling of Gelatinase Catalytic Domains and Docking Simulations of Novel Sulfonamide Inhibitors. *J. Med. Chem.* **1999**, *42*, 1723–1738, https://doi.org/10.1021/jm980514x.
14. Zhang, Y.-M.; Fan, X.; Xiang, B.; Chakravarty, D.; Scannevin, R.; Burke, S.; Karnachi, P.; Rhodes, K.; Jackson, P. Synthesis and SAR of α-sulfonylcarboxylic acids as potent matrix metalloproteinase inhibitors. *Bioorganic Med. Chem. Lett.* **2006**, *16*, 3096–3100, https://doi.org/10.1016/j.bmcl.2006.03.065.
15. Selivanova, S.V.; Stellfeld, T.; Heinrich, T.K.; Müller, A.; Krämer, S.D.; Schubiger, P.A.; Schibli, R.; Ametamey, S.M.; Vos, B.; Meding, J.; et al. Design, Synthesis, and Initial Evaluation of a High Affinity Positron Emission Tomography Probe for Imaging Matrix Metalloproteinases 2 and 9. *J. Med. Chem.* **2013**, *56*, 4912–4920, https://doi.org/10.1021/jm400156p.
16. Halder AK, Mallick S, Shikha D, Saha A, Saha KD, Jha TJRa. Design of dual MMP-2/HDAC-8 inhibitors by pharmacophore mapping, molecular docking, synthesis and biological activity. 2015;5:72373-86, https://doi.org/10.1039/C5RA12606A.
17. Adhikari, N.; Halder, A.K.; Mallick, S.; Saha, A.; Saha, K.D.; Jha, T. Robust design of some selective matrix metalloproteinase-2 inhibitors over matrix metalloproteinase-9 through in silico/fragment-based lead identification and de novo lead modification: Syntheses and biological assays. *Bioorganic Med. Chem.* **2016**, *24*, 4291–4309, https://doi.org/10.1016/j.bmc.2016.07.023.
18. Mukherjee, A.; Adhikari, N.; Jha, T. A pentanoic acid derivative targeting matrix metalloproteinase-2 (MMP-2) induces apoptosis in a chronic myeloid leukemia cell line. *Eur. J. Med. Chem.* **2017**, *141*, 37–50, https://doi.org/10.1016/j.ejmech.2017.09.052.
19. Ayoup MS, Fouad MA, Abdel-Hamid H, Abu-Serie MM, Noby A, Teleb MJEjomc. Battle tactics against MMP-9; discovery of novel non-hydroxamate MMP-9 inhibitors endowed with PI3K/AKT signaling attenuation and caspase 3/7 activation via Ugi bis-amide synthesis. 2020;186:111875, https://doi.org/10.1016/j.ejmech.2019.111875.

20. Beutel, B.; Song, J.; Konken, C.P.; Korpos, E.; Schinor, B.; Gerwien, H.; Vidyadharan, R.; Burmeister, M.; Li, L.; Haufe, G.; et al. New *in Vivo* Compatible Matrix Metalloproteinase (MMP)-2 and MMP-9 Inhibitors. *Bioconjugate Chem.* **2018**, *29*, 3715–3725, https://doi.org/10.1021/acs.bioconjchem.8b00618.
21. Wada CK, Holms JH, Curtin ML, Dai Y, Florjancic AS, Garland RB, et al. Phenoxyphenyl sulfone N-formylhydroxylamines (retrohydroxamates) as potent, selective, orally bioavailable matrix metalloproteinase inhibitors. 2002;45:219-32, https://doi.org/10.1021/jm0103920.
22. Breyholz, H.-J.; Schäfers, M.; Wagner, S.; Höltke, C.; Faust, A.; Rabeneck, H.; Levkau, B.; Schober, O.; Kopka, K. C-5-Disubstituted Barbiturates as Potential Molecular Probes for Noninvasive Matrix Metalloproteinase Imaging. *J. Med. Chem.* **2005**, *48*, 3400–3409, https://doi.org/10.1021/jm049145x.
23. Wang, J.; Medina, C.; Radomski, M.W.; Gilmer, J.F. N-Substituted homopiperazine barbiturates as gelatinase inhibitors. *Bioorganic Med. Chem.* **2011**, *19*, 4985–4999, https://doi.org/10.1016/j.bmc.2011.06.055.
24. Levin, J.I.; DiJoseph, J.F.; Killar, L.M.; Sung, A.; Walter, T.; Sharr, M.A.; Roth, C.E.; Skotnicki, J.S.; Albright, J. The synthesis and biological activity of a novel series of diazepine MMP inhibitors. *Bioorganic Med. Chem. Lett.* **1998**, *8*, 2657–2662, https://doi.org/10.1016/s0960-894x(98)00473-9.
25. Tamura, Y.; Watanabe, F.; Nakatani, T.; Yasui, K.; Fuji, M.; Komurasaki, T.; Tsuzuki, H.; Maekawa, R.; Yoshioka, T.; Kawada, K.; et al. Highly Selective and Orally Active Inhibitors of Type IV Collagenase (MMP-9 and MMP-2): *N*-Sulfonylamino Acid Derivatives. *J. Med. Chem.* **1998**, *41*, 640–649, https://doi.org/10.1021/jm9707582.
26. Cheng, M.; De, B.; Pikul, S.; Almstead, N.G.; Natchus, M.G.; Anastasio, M.V.; McPhail, S.J.; Snider, C.E.; Taiwo, Y.O.; Chen, L.; et al. Design and Synthesis of Piperazine-Based Matrix Metalloproteinase Inhibitors. *J. Med. Chem.* **2000**, *43*, 369–380, https://doi.org/10.1021/jm990366q.
27. Pikul, S.; Dunham, K.; Almstead, N.; De, B.; Natchus, M.; Taiwo, Y.; Williams, L.; Hynd, B.; Hsieh, L.; Janusz, M. HETEROCYCLE-BASED MMP INHIBITOR WITH P2'SUBSTITUENTS. **2001**, https://doi.org/10.2210/pdb1g05/pdb.
28. Levin J, Chen J, Du M, Hogan M, Kincaid S, Nelson F, et al. The discovery of anthranilic acid-Based MMP inhibitors. Part 2: SAR of the 5-position and P11 groups. 2001;11:2189-92.
29. Nelson, F.C.; Santos, E.D.; I Levin, J.; Chen, J.M.; Skotnicki, J.S.; DiJoseph, J.F.; A Sharr, M.; Sung, A.; Killar, L.M.; Cowling, R.; et al. Benzodiazepine inhibitors of the MMPs and TACE. *Bioorganic Med. Chem. Lett.* **2002**, *12*, 2867–2870, https://doi.org/10.1016/s0960-894x(02)00633-9.
30. Zask, A.; Gu, Y.; Albright, J.; Du, X.; Hogan, M.; Levin, J.; Chen, J.; Killar, L.; Sung, A.; DiJoseph, J.; et al. Synthesis and SAR of bicyclic heteroaryl hydroxamic acid MMP and TACE inhibitors. *Bioorganic Med. Chem. Lett.* **2003**, *13*, 1487–1490, https://doi.org/10.1016/s0960-894x(03)00127-6.
31. Aranapakam V, Davis JM, Grosu GT, Baker J, Ellingboe J, Zask A, et al. Synthesis and structure− activity relationship of N-substituted 4-arylsulfonylpiperidine-4-hydroxamic acids as novel, orally active matrix metalloproteinase inhibitors for the treatment of osteoarthritis. 2003;46:2376-96.
32. Le Diguarher, T.; Chollet, A.-M.; Bertrand, M.; Hennig, P.; Raimbaud, E.; Sabatini, M.; Guilbaud, N.; Pierré, A.; Tucker, G.C.; Casara, P. Stereospecific Synthesis of 5-Substituted 2-Bisarylthiocyclopentane Carboxylic Acids as Specific Matrix Metalloproteinase Inhibitors. *J. Med. Chem.* **2003**, *46*, 3840–3852, https://doi.org/10.1021/jm0307638.
33. Zhang, Y.-M.; Fan, X.; Chakaravarty, D.; Xiang, B.; Scannevin, R.H.; Huang, Z.; Ma, J.; Burke, S.L.; Karnachi, P.; Rhodes, K.J.; et al. 1-Hydroxy-2-pyridinone-based MMP inhibitors: Synthesis and biological evaluation for the treatment of ischemic stroke. *Bioorganic Med. Chem. Lett.* **2008**, *18*, 409–413, https://doi.org/10.1016/j.bmcl.2007.10.045.
34. Nuti, E.; Panelli, L.; Casalini, F.; Avramova, S.I.; Orlandini, E.; Santamaria, S.; Nencetti, S.; Tuccinardi, T.; Martinelli, A.; Cercignani, G.; et al. Design, Synthesis, Biological Evaluation, and NMR Studies of a New Series of Arylsulfones As Selective and Potent Matrix Metalloproteinase-12 Inhibitors. *J. Med. Chem.* **2009**, *52*, 6347–6361, https://doi.org/10.1021/jm900335a.
35. Becker DP, Barta TE, Bedell LJ, Boehm TL, Bond BR, Carroll J, et al. Orally active MMP-1 sparing α-tetrahydropyranyl and α-piperidinyl sulfone matrix metalloproteinase (MMP) inhibitors with efficacy in cancer, arthritis, and cardiovascular disease. 2010;53:6653-80, https://doi.org/10.1021/jm100669j.
36. Wilson, L.J.; Wang, B.; Yang, S.-M.; Scannevin, R.H.; Burke, S.L.; Karnachi, P.; Rhodes, K.J.; Murray, W.V. Discovery of novel Cobactin-T based matrix metalloproteinase inhibitors via a ring closing metathesis strategy. *Bioorganic Med. Chem. Lett.* **2011**, *21*, 6485–6490, https://doi.org/10.1016/j.bmcl.2011.08.068.
37. Topai, A.; Breccia, P.; Minissi, F.; Padova, A.; Marini, S.; Cerbara, I. In silico scaffold evaluation and solid phase approach to identify new gelatinase inhibitors. *Bioorganic Med. Chem.* **2012**, *20*, 2323–2337, https://doi.org/10.1016/j.bmc.2012.02.010.
38. Hugenberg, V.; Breyholz, H.-J.; Riemann, B.; Hermann, S.; Schober, O.; Schäfers, M.; Gangadharmath, U.; Mocharla, V.; Kolb, H.; Walsh, J.; et al. A New Class of Highly Potent Matrix Metalloproteinase Inhibitors Based on Triazole-Substituted Hydroxamates: (Radio)Synthesis and in Vitro and First in Vivo Evaluation. *J. Med. Chem.* **2012**, *55*, 4714–4727, https://doi.org/10.1021/jm300199g.

39. Kreituss, I.; Rozenberga, E.; Zemītis, J.; Trapencieris, P.; Romanchikova, N.; Turks, M. Discovery of aziridine-triazole conjugates as selective MMP-2 inhibitors. *Chem. Heterocycl. Compd.* **2013**, *49*, 1108–1117, https://doi.org/10.1007/s10593-013-1351-9.
40. Scannevin RH, Alexander R, Haarlander TM, Burke SL, Singer M, Huo C, et al. Discovery of a highly selective chemical inhibitor of matrix metalloproteinase-9 (MMP-9) that allosterically inhibits zymogen activation. 2017;292:17963-74, https://doi.org/10.1074/jbc.M117.806075 .
